# Insight into SNPs and epitopes of E protein of newly emerged genotype-I isolates of JEV from Midnapur, West Bengal, India

**DOI:** 10.1186/s12865-017-0197-9

**Published:** 2017-03-06

**Authors:** Shyamashree Banerjee, Parth Sarthi Sen Gupta, Amal Kumar Bandyopadhyay

**Affiliations:** Department of Biotechnology, The University of Burdwan, Burdwan, West Bengal 713104 India

**Keywords:** Japanese encephalitis virus, Genotype I, Genotype III, Midnapur, Homology model, SNP Energetics, B-cell & T-cell epitopes, PEP-FOLD, Docking

## Abstract

**Background:**

Japanese encephalitis virus (JEV) is a mosquito-borne flavivirus that causes Japanese Encephalitis (JE) and Acute Encephalitis Syndrome (AES) in humans. Genotype-I (as co-circulating cases with Genotype-III) was isolated in 2010 (JEV28, JEV21) and then in 2011 (JEV45) from Midnapur district, West Bengal (WB) for the first time from clinical patients who were previously been vaccinated with live attenuated SA14-14-2 strain. We apply bioinformatics and immunoinformatics on sequence and structure of E protein for analysis of crucial substitutions that might cause the genotypic transition, affecting protein-function and altering specificity of epitopes.

**Results:**

Although frequency of substitutions in E glycoprotein of JEV28, JEV21 and JEV45 isolates vary, its homologous patterns remain exactly similar as earlier Japan isolate (Ishikawa). Sequence and 3D model-structure based analyses of E protein show that only four of all substitutions are critical for genotype-I specific effect of which **N103K** is common among all isolates indicating its role in the transition of genotype-III to genotype-I. Predicted B-cell and T-cell epitopes are seen to harbor these critical substitutions that affect overall conformational stability of the protein. These epitopes were subjected to conservation analyses using a large set of the protein from Asian continent.

**Conclusions:**

The study identifies crucial substitutions that contribute to the emergence of genotype-I. Predicted epitopes harboring these substitutions may alter specificity which might be the reason of reported failure of vaccine. Conservation analysis of these epitopes would be useful for design of genotype-I specific vaccine.

**Electronic supplementary material:**

The online version of this article (doi:10.1186/s12865-017-0197-9) contains supplementary material, which is available to authorized users.

## Background

Japanese encephalitis virus (JEV), a mosquito-borne flavivirus of the family Flaviviridae, is the sole etiologic agent of Japanese encephalitis (JE). JE is the neurotropic disease has been the primary health concern in human worldwide mostly affecting children and older person. Approximately 25–30% of JEV cases are fatal and 50% result in permanent neuropsychiatric sequelae [1, 2].

In India, JE was first reported in Vellore in 1955 [1, 2]. Since then, epidemics of JE in different states have been reported including large and severe outbreak in the State of Uttar Pradesh (UP) and part of Bihar that caused 5737 JE cases and 1,334 deaths in UP and 360 cases with 64 deaths in Bihar in 2005 [1]. Analyses showed that genotype III (GIII) is predominant in India. However, genotype I (GI) has recently been introduced in different States in India including West Bengal [2]. Since 1973, JE outbreaks have been recorded in different districts of West Bengal (WB). Although, The State Health Department, Govt. of WB routinely conducts vaccination program (live attenuated SA14-14-2, GIII) against JE cases in different districts of WB, sporadic JE cases and deaths are being reported every year. The vaccination program is challenged by the repeated observation of co-circulation cases of GIII and GI in prevaccinated patients in the district of Midnapur of WB [3, 4].

Viral genome produces single polyprotein which is processed by viral and host proteases into structural (capsid C, precursor membrane prM and envelope E) and non-structural (NS1, NS2A, NS2B, NS3, NS4A, NS4B and NS5) proteins [5, 6]. Although all these proteins are important for viral life cycle, the E protein is responsible for host cell interaction, infectivity and entry of the virus [6]. Amino acid sequence of full length genome of virulent wild-type (WT) JEV strain (SA14) when compared with that of non-virulent live-attenuated SA14-14-2 vaccine strain, it was found that a multitude of mutations are accumulated throughout the genome of the later. These genome wide mutations are collectively considered to be responsible for non-virulent property of SA14-14-2. E protein accumulates maximum number of mutations in the attenuation process and thus it is considered as the primary genetic determinant for neurovirulence and neuroinvasiveness of WT strains of JEV [5, 7]. However, detailed mechanism for neurovirulence and neuroinvasiveness in relation to locus specific amino acid changes is unknown.

E protein based phylogenetic analyses have established five different genotypes of JEV of which GIII constitutes the major genotype in Asian countries including China, Japan, Taiwan, India, Korea, Thailand, Indonesia, and Vietnam. Genotyping is commonly achieved by construction of phylogenetic tree using E protein of isolates and obtaining clades [8]. Although in low frequency, GI is seen to replace the existing GIII from all these countries [9]. In India like other countries, GIII is the dominant genotype for all states. However, co-circulation of GI with GIII in AES/JE patients was first documented from Gorakhpur/UP/India region in 2009 [9] and then from Midnapur district of West Bengal (WB), India in 2010 and 2011 [3, 4]. In the later case emergence of GI with GIII was observed in patient even when it was reported to be immunized with SA14-14-2 vaccine prior infection [4, 10]. Similar observation is also available from China [11].

In general the sequence of E protein of virulent isolate of JEV is compared with that of non-virulent SA14-14-2 to gain insight into neurovirulence and neuroinvasiveness effects of the former. While high neurovirulence of WT JEV isolates was reported to be contributed by eight mutations (**F107L, K138E, V176I, A177T, H264Q, M279K, V315A** and **R439K**) in E protein, neuroinvasiveness involves mutations in other proteins also [12–15]. Notably both GI (JEV45) and GIII (JEV46 and JEV47) isolates of WB were demonstrated to be neurovirulent and possess the above mutations in E protein [4]. Additional substitutions observed in these isolates were claimed to have potential for i] altering immunogenicity [based on HLA (Human leukocyte antigen) class I specific decreased/increased binding scores] and ii] escape of antibody neutralization based on their occurrence in known loop structures of E protein of West Nile Virus and decreased/increased hydrophilicity values [4]. However, the question that which among all mutations may have relation with the observed genotypic transition, alteration of epitope’s specificity and E protein function remains to be worked out. Reported failure of conventional vaccination and emergence of GI in the region needs careful prediction of B-cell and T-cell epitopes to improve immunization in the locality at high risk of disease.

In the present study we consider E protein of newly emerged GI isolates from Midnapur/WB to develop homology model structures possessing either all or single mutation. We then use both mutant structure and sequence based methods to identify critical among all acquired substitutions. Linear as well as conformational B-cell and T-cell (HLA class I and II specific) epitopes are also been worked out. We further report improved filtering of epitopes using conservation, accessibility, conformational stability and docking criteria. Using large database of E protein from Asian countries, genotypic diversity of the epitopes is established in this study. Taken together, the study identifies GI specific critical SNPs and epitopes and finds application in immunoinformatics.

## Methods

### Protein sequence retrieval and analysis

Full length nucleotide sequences of E protein that are isolated from Midnapur district, WB, India as GI were retrieved from GenBank database [ID: JN703381 (JEV28/2010); JN703382 (JEV21/2010); KC526872 (JEV45/2011)]. We also procured Ishikawa/Japan GI [ID: AB051292] for comparison. The nucleotide sequences were converted into proteins using EMBOSS (European Molecular Biology Open Software Suite) Transeq tool [16]. Primary structure analysis was performed using our laboratory procedures PHYSICO and PHYSICO2 [17, 18]. Secondary structure analysis was performed using SOPMA [19].

### Homology modeling, energy minimization, evaluation and submission

Homology modeling, energy minimization and evaluation have been routine procedures for many proteins [20–23]. Crystal structure of JEV ecto domain of E protein of SA14-14-2 (GIII) is available in the Protein Data Bank (PDB) with ID 3P54.pdb (Chain A, Resolution 2.1 Å). The PDB sequence (UNIPROT ID: P27395) has 97.3, 96.3, 96.3 and 95.3% identity with that of JEV28 (JN703381), JEV21 (JN703382), JEV45 (KC526872) and Ishikawa (AB051292) isolates respectively. There is no INDEL region for any of these sequences when aligned with reference template sequence (P27395). We therefore performed homology modeling on the target sequences using 3P54_A as template. Comparative model of the target sequences were achieved by MODELLER 9 v11 package [24] that makes use of spatial restraints and other optimization procedures. Alignments between template and target sequences were performed by using MODELLER in-built script. Manual improvements were incorporated in the alignment when it was necessary. At least five replicates of model were produced of which the best model was selected based on Discrete Optimized Protein Energy (DOPE) [25] assessment scores. Idealization of bond geometry, optimization of loop regions and removal of unwanted non-bonded contacts of the initial model was achieved by energy minimization using CHARMM (Chemistry at HARvard Macromolecular Mechanics) force field of NAMD (Nanoscale Molecular Dynamics) package [26]. To retain known disulfide bond specificity, protein's residue pairs are appropriately patched. Explicit water box of 5 Å additional distance from the highest coordinate in each dimension with an exclusion radius 2.4 Å was incorporated with the model using VMD (Visual molecular dynamics) in-built solvate v1.5 plugin [27] and subjected for minimization. After 5000 steps of conjugate gradient minimization, trajectory analyses were performed by using Vega ZZ interface [28] to select the lowest energy frame of models. The resulted models were then subjected for a series of tests such as stereo-chemical analyses by PROCHECK [29] geometric relationship among non-bonded atoms by ERRAT [30], proper threading of target sequence with the model structure by VERIFY3D [31] and structural features based overall quality by ProQ. [32]. Ion-pair specificity and energetics of models and template were compared using SBION [33], SBION2 [34] and ADSBET2 [35]. High quality models thus obtained were deposited in the Protein Model Data Base (PMDB) [36] and the PMDB identifiers were assigned as **PM0080323, PM0079295, PM0080324** and **PM0080325** for JEV28, JEV21, JEV45 and Ishikawa respectively.

We also developed model for each positional mutation observed in GI's E protein. In this procedure, we first generated target sequence from template (3P54_A) by giving one observed mutation at a time. The derived target sequence was then used for model development as described. Each model was evaluated as mentioned above. These mutant structures were used along with the template (WT) to evaluate the effect of mutation (see below).

### Disease related SNP prediction

The single nucleotide polymorphism occurring in the protein coding region may lead to deleterious consequences and might affect epitopes conformational properties in protein. In order to identify disease-associated SNPs, we used SIFT [37], PhD-SNP [38] SNAP [39] and META-SNP [40] authentic web server for sequence based and SDM (Site Directed Mutator) [41] web server for structure based prediction. SIFT prediction is based on the sequence homology and the physicochemical properties of amino acids which are dictated by the substituted amino acid. PhD-SNP uses support vector machines method based human deleterious SNP. It predicts whether the given amino acid substitution leads to disease associated or neutral. META-SNP is a high accuracy integrated method to discriminate between disease-related and polymorphic non-synonymous single nucleotide variants. SDM is a statistical method for calculation of difference in free energy between wild type and mutant protein and prediction of disease association.

### B-cell and T-cell epitope prediction

Prediction of both B cell and T cell epitopes is the first step for rational vaccine design. Sequences of E protein of GI isolates (Ishikawa, JEV28, JEV21 and JEV45) were used for prediction of B-cell linear epitopes using BCPREDS v1.0 [42] web server. Epitopes thus obtained were subjected for further screening based on various criteria such as i] model energy [43], ii] average side chain accessibility using NACCESS procedure [44], iii] Shannon Entropy based conservation properties [17, 18] and iv] chances to harbor critical substitutions of epitopes. Finally only 8 epitopes were selected.

Conformational B cell epitopes are determined using high accuracy web server EPSVR [45] using model 3D structures as input. The method uses support vector regression for prediction. The result file represents residues in color codes from low (blue i.e. ≤40) to high (red i.e. ≥90). Only the high possibility residues (≥80%) are highlighted in the model structure using VMD interface [27].

Cellular peptide vaccine contains T-cell epitopes that bind structurally to highly diverse, polygenic and polymorphic human MHCs (Major histocompatibility complex) whose experimental determination are cost effective and time consuming. IEDB analysis tools [46] were used for MHC class specific (class I and II) T cell epitopes prediction. All available MHC class specific HLA-alleles were tested for their efficacy in binding epitopes under the strict cutoff of percentile rank (equivalent to IC_50_ value). Clustering of alleles against each top score epitope was performed in excel. Epitopes were further short listed by their antigenicity scores using VaxiJen v2.0 server [47]. MHC class I epitopes were further screened based on proteosomal processing score. Each set of epitopes were further short listed using average conservation [17, 18] and accessibility [44] value. Finally only best 10 epitopes from each class (MHC - I and MHC - II) were retained along with their respective HLA alleles.

### Structure prediction of epitopes and docking study

Docking of T-cell class specific epitope on known binding site (PBG: peptide binding groove) of respective MHC class specific PDB structure was performed for further short listing. Optimized epitope structure was obtained using HMM-SA (Hidden Markov Model-derived Structural Alphabet) [48] with OPEP (Optimized Potential for Efficient protein structure Prediction) force field [49]. 2X4O and 1DLH were used as peptide targets for MHC class I and II respectively. Both these targets were in complex form with inhibitors. Structure with single binding site and free from inhibitor was procured using VMD interface [27]. Binding sites of each target was mapped in presence of inhibitor using NACCESS procedure [44]. Docking of above epitopes (10 from each class) onto its respective targets (2X4O and 1DLH) were performed using PatchDock server [50] along with input of computed binding site. Epitope-receptor complex was then refined using FireDock [51] server. Best four epitopes from each class were selected on the basis of energetics and interaction criteria.

## Results

Like other countries in Asia [2, 8], JEV mediated acute encephalitis syndrome and associated damage and death in patients are the major health concern in India in different states including WB [3, 4, 9]. The report of co-circulation of GI with GIII in the district of Midnapur of WB increases the risk of possible outbreak in the locality. The appearance of the former was documented even when Government aided regular vaccination program was in vogue [3]. Such threatening genotypic transition and failure of neutralization raise the possibility of critical mutations in envelope glycoprotein. In this study we therefore investigate substitutions in E protein of GI strains (Ishikawa, JEV28, JEV21 and JEV45) in reference to the GIII vaccine strain (SA14-14-2).

### E protein of GI strain acquires domain specific substitutions

Which substitutions contribute to the transition from existing GIII to emerging GI? To check this we have presented observed substitutions in E protein of GI isolates in Table 1 along with reference GIII strains. Ishikawa, a typical GI isolate that caused outbreak in Japan in 1994 and possesses similar mutations at homologous positions as WB isolates is also included for comparison purpose. Six mutations: **K138E, V176I, A177T** in Domain I and **F107L, H264Q, M279K** in domain II of E protein (Table 1, marked with ^a^ sign) are common in all the GI isolates. They are also present in virulent GIII strain SA14 (Table 1: column 8) from which the live attenuated non-virulent SA14-14-2 strain was derived. Notably, north India epidemic (in 2005) strain GP78 (ID AF075723) [1] and recent GIII isolates (JEV46 and JEV47) from WB [4] also contain the above six mutations. As these mutations in E protein are common in SA14, GI and GIII virulent isolates, net mutations in our GI isolates are counted without them. However following points are noteworthy from the Table 1: i] WB isolates have similar positional substitutions as earlier Japan isolate (Ishikawa) which has highest number of mutations in all three domains of E protein, ii] JEV28 (2010) has fewer mutations in most immunogenic domain III than JEV21 (2010) and JEV45 (2011). Substitutions **V372L, M374I, G388K, W396R** are absent in JEV28, iii] four amino acid changes in PDB (3P54_A) sequence relative to UNIPROT one (ID P27395): **K138E, V176I, F107L** and **M279K** were considered as mismatch [52].

**Table 1:** List of domain specific mutations in E protein of GI isolates in reference (Ref) to GIII strains

		Genotype I (GI)				Genotype III (GIII)		
E Protein	Mutations	AB051292 [Japan, Ishikawa, 1994]	JN703381 [WB, JEV28 2010]	JN703382 [WB, JEV21 2010]	KC526872 [WB, JEV45 2011]	3P54	SA14 virulent M55506	SA14-14-2 Non-virulent JN604986
Domain I	N--2H	.	+	+	+	N	N	N
	N--2 T	+	.	.	.	N	N	N
	Y183F	+	.	.	.	Y	Y	Y
	R193K	+	.	.	.	R	R	K
	K138E^a^	(+)^a^	(+)^a^	(+)^a^	(+)^a^	K^b^	E	K
	V176I^a^	(+)^a^	(+)^a^	(+)^a^	(+)^a^	V^b^	I	V
	A177T^a^	(+)^a^	(+)^a^	(+)^a^	(+)^a^	T	T	A
Domain II	C-60Y	+	.	.	.	C	C	C
	N103K	+	+	+	+	N	N	N
	F107L^a^	(+)^a^	(+)^a^	(+)^a^	(+)^a^	F^b^	L	F
	T129M	+	.	.	.	T	T	T
	A222S	+	+	+	+	A	A	A
	G244E	+	+	+	+	G	G	G
	G261S	.	+	+	+	G	G	G
	H264Q^a^	(+)^a^	(+)^a^	(+)^a^	(+)^a^	Q	Q	H
	M279K^a^	(+)^a^	(+)^a^	(+)^a^	(+)^a^	M^b^	K	M
Domain III	V315A	+	+	+	+	V	V	V
	S327T	+	+	+	+	S	S	S
	A366S	+	.	.	.	A	A	A
	V372L	+	.	+	+	V	V	V
	M374I	+	.	+	+	M	M	M
	G388K	+	.	+	+	G	G	G
	W396R	+	.	+	+	W	W	W
Total mutations		21	13	17	17	4	6	Ref
Net mutations		15	7	11	11	-	-	

Amino acids marked with ^b^sign in 3P54 have been identified as mismatches between PDB and UNIPROT sequence (P27395)

All comparisons are made for first 406 residues of E protein (ecto domain). All three GI isolates of Midnapur/WB are taken with earlier Japan strain (Ishikawa). In GIII category, three sequences such as 3P54_A, virulent SA14 (ID M55506) and attenuated non-virulent SA14-14-2 (ID JN604986) are considered. + Sign indicates presence and dot indicates absence of mutations respectively. (+) indicates category of reported neurovirulent mutations [13]. Identical neurovirulent mutations of present isolates as SA14 are identified with^a^ sign

**Fig. 1:**
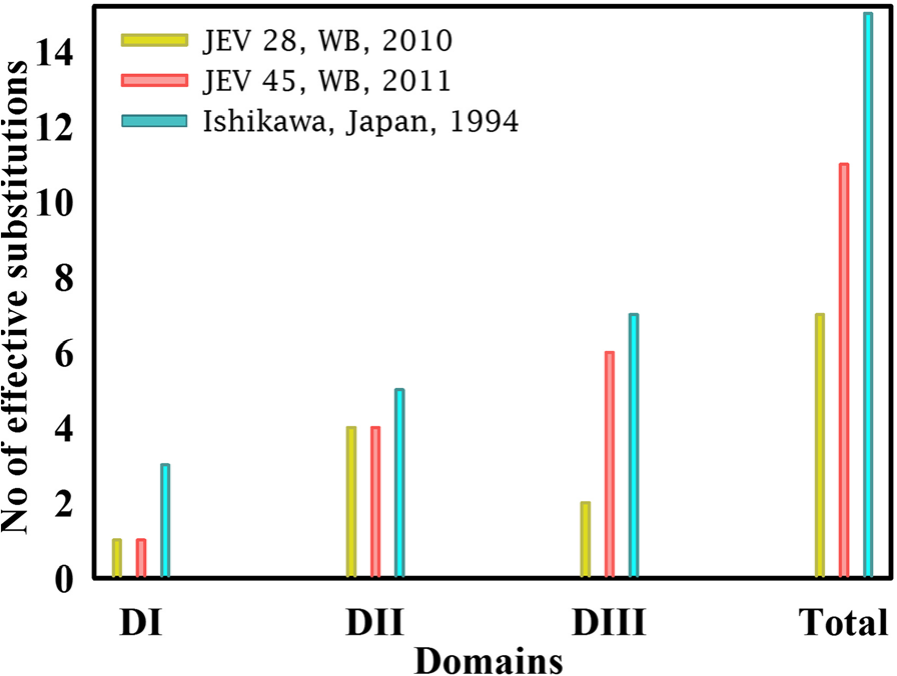
Comparison of net substitutions for each domains and total of E protein among JEV28 (*yellow*), JEV45 (*red*) and Ishikawa isolates (*cyan*). JEV21 is not included in the plot as it is identical to JEV45

To check domain distribution of net substitutions (Table 1) of E protein, we have presented the following Figure (Fig. 1). Several points are noteworthy from the Figure. Firstly as we move from DI to DIII, substitution frequency increases both for Ishikawa and JEV45 isolates. The later, just one year younger than JEV28/2010, is seen to acquire almost identical loci specific substitutions as Ishikawa/Japan/1994. Secondly, as far as total substitutions are concerned, Ishikawa dominates over both JEV28 and JEV45. Finally, relative to JEV28 three additional substitutions in JEV45 are seen in domain-III. The domain is reported to be most immunogenic [53].

### Structural insight into E proteins of GI isolates

**Fig. 2:**
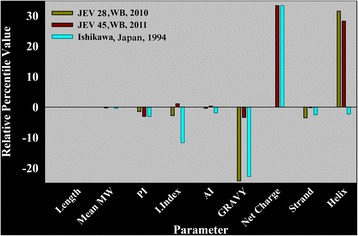
Residual plot of average physicochemical properties for GI isolates (Ishikawa, JEV28 and JEV45) in reference to SA14-14-2. Analyses were performed using PHYSICO [17] and PHYSICO2 [18] programs with an input of E proteins of SA14-14-2, Ishikawa, JEV28 and JEV45 in FASTA format. Percentile difference for each property was computed against the reference data and plotted using SYSTAT Sigma Plot v11.0

The ecto domain of E protein is composed of 406 residues that contains majority of the antigenic determinants. The sequence identity of E protein of Ishikawa, JEV28 and JEV45 are 94.8, 96.8 and 95.8% respectively relative to SA14-14-2. Primary and secondary structural properties of E protein of these GI isolates (Ishikawa, JEV28 and JEV45) are compared with reference sequence (SA14-14-2) in Fig. 2. It is seen that most of physicochemical properties, except GRAVY (Grand Average Hydropathy), net charge and helix content remain almost similar as reference. These properties show modulation among GI isolates. For example, while in Ishikawa and JEV28, GRAVY decline, net charge of Ishikawa and JEV45 show an increase. Although Ishikawa remains unaffected, coil to helix transition is apparent for both JEV28 and JEV45.

Homology modeling allows gaining structural insight from protein sequence [54] which further helps to extract structural information [55, 56] including prediction of conformational epitopes [57], localizing linear epitopes [42] and prediction of the effects of SNPs via thermodynamic cycle of folded and unfolded states of wild and mutant proteins [58]. In present study, we developed a total of 18 model structures (3 plus 15) for ecto domain of E protein. The first three models are of E protein of Ishikawa, JEV28 and JEV45 isolates. The rest 15 are models wherein each structure possesses single positional mutation (as in Table 1) with reference to the vaccine strain, SA14-14-2. While the former three are useful for general structural characterization of substitutions and epitopes, the later are crucial for understanding the effect of SNP on overall conformational stability of protein, and its disease association in relation to protein function. Homology models were obtained by the use of advance modules of Modeller v9.11 [24] followed energy minimization using CHARMM force field of NAMD package [26] in presence of explicit water as solvent with appropriate disulfide bond patching. Models thus obtained were evaluated as earlier procedures [20–23]. Topology of each model was compared with that of template by superposition of C_α_ atomic coordinates and the average RMSD (Root mean square deviation) value was found to vary in the range 1.12–1.44 Å. Absolute per residue sum of surface area by NACCESS [44] procedure for template is seen to be 40.1 Å^2^ and that for models (Ishikawa, JEV28 and JEV45) vary between 42.9 and 43.7 Å^2^. In the former, frequency of buried residues is seen to be 161 and that for models vary between 160 and 161 when threshold for core is set to ≤ 20 Å^2^ value. Detailed comparison between template and models on salt bridges and its energetics were performed using our laboratory developed software [33–35]. Frequency of salt bridges (19 to 23) of models and side-chain specificity of their partners remain almost similar (≥75%) as template (data not shown).

**Fig. 3:**
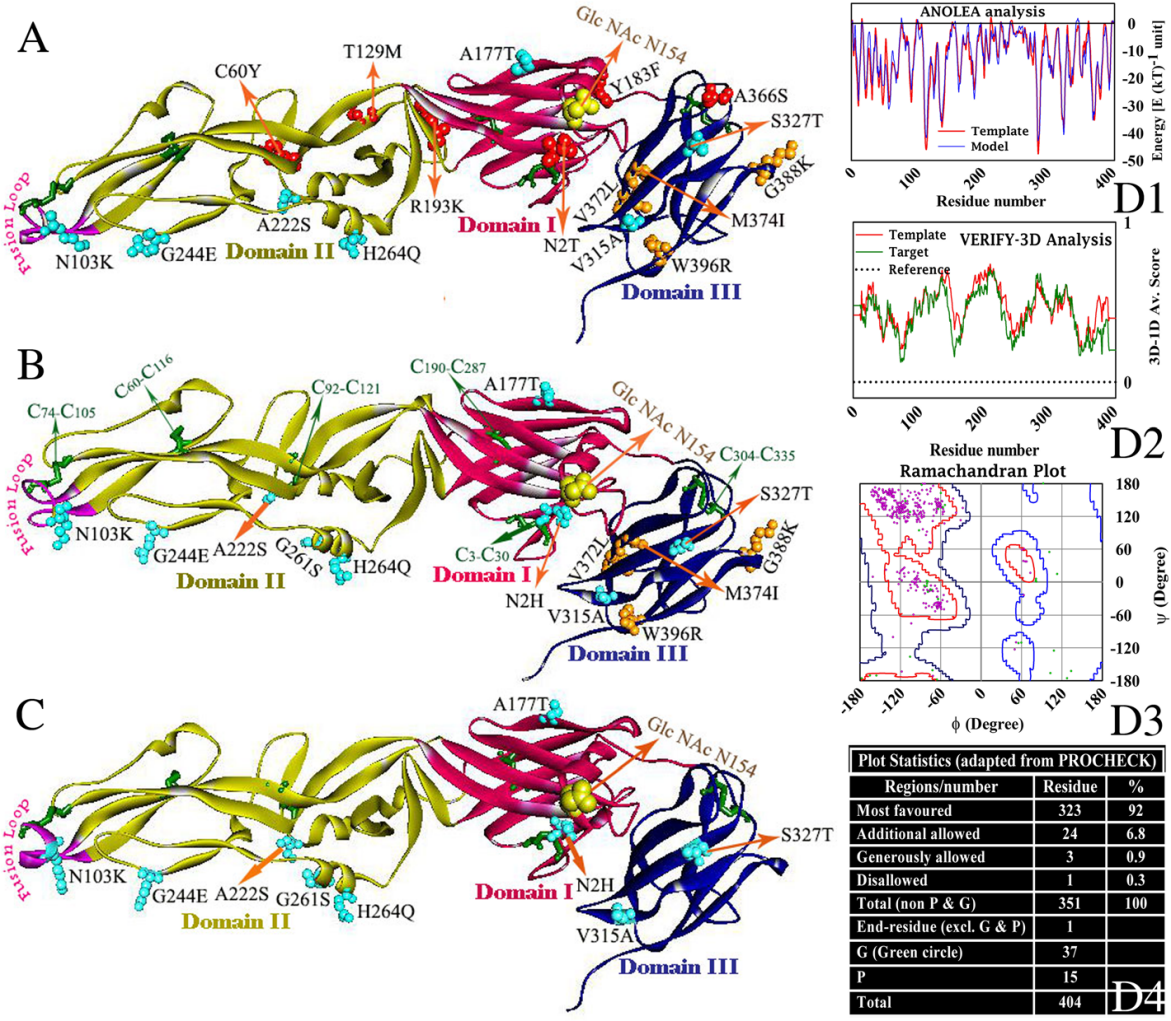
Backbone Cα-traces of homology model (**a** for Ishikawa, **b** for JEV45 and **c** for JEV28) of the ecto domain of **PM0080325**, **PM0080324** and **PM0080323** respectively. Domain I (*red*), domain II (*yellow*) and domain III (*blue*) are shown in different colors. Fusion loop (*purple*) and substitutions with respect to the template (SA14-14-2) are highlighted in each of the model based on their occurrence (see Table 1). Disulfide bonds (*green*) are also highlighted in each of the model based on their occurrence and only labeled in case of JEV45. Comparison of ANOLEA-profiles [61] of **PM0080324** (*green* trace) with template (*red* trace) (D1), VERIFY3D (D2) analysis [31] and Ramachandran plot (D3) of main chain dihedral angles (core region is outlined in *deep blue* and allowed region in *red*) for residues (glycine as *green* circle and non-glycine as *pink* points) of the model along with PROCHECK [29] analysis (D4) are presented for model validation

Model structure of Ishikawa, JEV45 and JEV28 are presented in Fig. 3a, b and c respectively. Each of these structures highlights substituted residues (Table 1), disulfide bonds and domains (as DI, DII and DIII). Domain I is the middle domain that (Fig. 3, red colored region reconstituted by 127 residues in three stretches: 1–51, 135–193 and 283–299) possesses nine stranded β-barrel structure with two disulfide bonds (***C***_***3***_ ***-C***_***30***_ and ***C***_***190***_ ***-C***_***287***_) and model specific amino acid substitutions (**N2H, N2T**, **A177T, Y183F** and **R193K**). It also contains the conserved glycosylation site (**N154**). Domain II (yellow colored) is the distal domain which forms extended structure of 172 residues constituted by two regions (52–134 and 194–282) of E protein. It is stabilized by three disulfide bonds (***C***_***60***_ ***-C***_***116***_ *,* ***C***_***92***_ ***-C***_***121***_ and ***C***_***74***_ ***-C***_***105***_). This domain contains a total of seven substitutions (see Table 1) with respect to the template (**C60Y, N103K** ***,*** **T129M, A222S**, **G244E** ***,*** **G261S** and **H264Q**). Fusion loop which is 13 residues long is shown by purple color (at tip of Domain II) is needed for pathogenesis, infectivity and broad range antibody cross-reactivity [52, 59]. It is highly conserved among all flaviviruses. Domain III (blue colored globular domain) of the model contains 100 residues (300–399) and forms typical immunoglobulin like fold at the C-terminus of the ecto domain of E protein. This domain possesses one disulfide bond (***C***_***304***_ ***-C***_***335***_) and seven substitutions (**V315A, S327T, A366S, V372L, M374I, G388K** and **W396R**). In this domain a tripeptide sequence **RGD** (387–389; blue colored) form a motif which is believed to play role in receptor interaction [5, 6, 60].

Although all models were evaluated using multiple authentic procedures [20–23], results for the model JEV45 is shown in the Right panel of the Fig. 3. Energetic profile of the model (green trace) and the template (red trace) are seen to be almost identical when plotted as a function of residue position as obtained by ANOLEA [61] (Fig. 3: D1) and VERIFY3D analysis [31] (Fig. 3: D2). Ramachandran plot for main chain dihedral angles and PROCHECK analysis [29] (Fig. 3: D3 and D4 respectively) show amino acid residues, occupying core (92%) and allowed (8%) regions.

### Disease relation of substitutions

There are a maximum of 15 substitutions for GI isolates apart from six reversal type (Tables 1 and 2). Are all these substitutions lethal? How could they be related with protein function and disease association? To resolve this, we present results of sequence and structure based prediction of the effect of these SNPs in Table 2. Sequence based prediction identify fatal substitutions as D and normal as N based on score. Structure based method computes overall conformational free energy change (***ΔΔG***_***WT***→***Mu***_) in Kcal mol^−1^. These results (Table 2) show that only 4 of 15 mutations are lethal, disease associated and cause protein malfunctioning. Rest 11 substitutions are normal and non-disease associated. In this group of mutations, +0.50 ≤ **ΔΔG ≤** +2.00 and −2.00 ≤ **ΔΔG ≤ −**.50 categories are also observed.

**Table 2:** Sequence and structure based evaluation of the effect of mutations (Table 1)

		Sequence based prediction								Structure based prediction
	Mutation	SIFT		PHD-SNP		SNAP		META-SNP		SDM
		Score	Effect	Score	Effect	Score	Effect	Score	Effect	∆∆G Kcal Mol^−1^
Domain I	N002H	0.500	N	0.056	N	0.180	N	0.115	N	+0.01
	K138E^a^	1.000	N	0.121	N	0.185	N	0.111	N	+0.52
	V176I^a^	0.400	N	0.047	N	0.160	N	0.090	N	+0.22
	A177T^a^	1.000	N	0.042	N	0.105	N	0.063	N	−0.17^b^
	Y183F	0.200	N	0.488	N	0.525	N	0.367	N	+1.79
	R193K	0.450	N	0.490	N	0.340	N	0.414	N	−0.81
Domain II	C060Y	0.000	D	0.935	D	0.800	D	0.882	D	−2.33
	N103K	0.000	D	0.833	D	0.780	D	0.771	D	+1.61
	F107L^a^	1.000	N	0.218	N	0.310	N	0.155	N	−0.97
	T129M	0.070	N	0.106	N	0.410	N	0.143	N	+0.86
	A222S	0.420	N	0.177	N	0.115	N	0.162	N	−0.77
	G244E	1.000	N	0.070	N	0.215	N	0.065	N	+1.85
	G261S	0.200	N	0.152	N	0.105	N	0.134	N	+1.74
	H264Q^a^	0.074	N	0.074	N	0.225	N	0.078	N	−0.75^b^
	M279K^a^	0.390	N	0.159	N	0.255	N	0.121	N	−1.97
Domain III	V315A	0.620	N	0.066	N	0.400	N	0.094	N	−1.63
	S327T	0.820	N	0.067	N	0.175	N	0.103	N	+0.97
	A366S	0.740	N	0.190	N	0.235	N	0.185	N	−1.18
	V372L	0.310	N	0.129	N	0.245	N	0.164	N	−0.79
	M374I	0.540	N	0.225	N	0.365	N	0.169	N	+0.08
	G388K	0.010	D	0.910	D	0.775	D	0.793	D	+3.00
	W396R	0.000	D	0.841	D	0.865	D	0.819	D	−2.48

*D* disease, *N* normal; ^a^indicates these six mutations are not GI specific but also present in SA14, GIII isolates with reference to vaccine strain SA14-14-2 (Table 1);

^b^indicates ∆∆G was calculated in reverse mutation form i.e. T177A and Q264H as WT E protein possesses T and Q at these positions respectively Server based four independent methods for sequence of ecto domain of E protein and Site Directed Mutator (SDM) [41] method for structure of all isolates was used for the purpose (see Materials and Methods for details)

One of the fatal substitutions (from sequence based methods) i.e. **N103K** is seen to be common in all GI isolates (Table 1). It is present in the fusion loop region (Fig. 3) which is known to initiate host-virus interaction and eventual viral entry. Two of the fatal substitutions i.e. **G388K** and **W396R** are common for JEV21, JEV45 and Ishikawa but absent in JEV28. Both these substitutions are present in antigenic domain III of E protein (Fig. 3). The substitution **C60Y** is only present in Ishikawa/Japan isolate but not in any of the WB isolates. Notably **C60** is involved in the formation of disulfide bond in domain II. Unlike normal, these 4 fatal substitutions show high change of overall conformational free energy of which **G388K** and **N103K** are positive and that in case of **W396R** and **C60Y** are negative.

### Epitope prediction

Envelope glycoprotein of JEV is 500 amino acids long of which ecto domain constitutes about 406 residues. The protein has been the major focus for immunoinformatics studies for its neutralizing activity and antigenic cross reactivity from different flaviviruses [62, 63]. In fact clathrin-mediated viral internalization was reported to be guided by the protein. At present the only available vaccine for prevention of JEV mediated AES/JE is derived from live or inactivated form of GIII strain SA14-14-2. However, the efficacy of immunization with the current vaccine was questioned due to the fact that prevaccinated patients showed symptoms of JE/AES with co-circulation of GI strain in their serum [4, 10]. Such reports of emergence of GI strain from the pool of GIII in Asian countries signaling for design of high selective epitopes.

### B-cell epitope prediction

B-cell epitopes are effective for induction of neutralizing antibody in relation to the viral entry. Identification and characterization of these epitopes would help in design of vaccine. B-cell epitopes having high prediction score, low model energy (i.e. high conformational stability), high average accessibility to the surface of protein and high average conservation were selected (Fig. 3). Our predicted epitopes (Table 3) show overlap with predetermined epitope segments [64]. 7 of 8 epitopes (Table 3) seen to harbor GI specific substitutions (Table 1) and four of these seven epitopes namely VEMEPPFGDSYIVVGR**G**DKQ, GWG**K**GCGLFGKGSIDTCAKF, H**W**HKAGSTLGKAFSTTLKGA and IEASQLAEVRSY**Y**YHASVTD are seen to contain fatal substitutions.

**Table 3:** B cell specific antigenic peptide epitopes short listed from a large set of initial population based on their antigenic score, model energy, average ASA (Accessible surface area) and average conservation

Start Position	Linear Epitope	Score	Model Energy	Av. ASA	Av. H
325	EL**T**YSGSDGPCKIPIVSVAS	0.986	−23.71	22.8	0.01
372	VEMEPPFGDSYIVVGR**G**DKQ	0.977	−24.54	37.1	0.02
100	GWG**K**GCG**L**FGKGSIDTCAKF	0.956	−29.81	42.1	0.02
69	STVARCPTTGEAHNEKRADS	0.926	−12.95	59.3	0.01
395	H**W**HKAGSTLGKAFSTTLKGA	0.876	−23.38	97.2	0.02
121	CTSKAIGRMIQPENIKY**E**VG	0.854	−18.21	38.2	0.06
349	TPVGRLVTVNPFVATSS**S**NS	0.923	−20.55	39.9	0.04
48	IEASQLAEVRSY**Y**YHASVTD	0.967	−32.65	31.4	0.06

NACCESS program [44] was used to generate residue accessibility data. Then average accessibility was computed using side chain relative accessibility values. Variability was calculated using full length E protein sequences (total 50) and positional Shannon entropy (**H**) [17, 18] was computed for each homologous position. The Av. Variability is the average of all residues value. Average is made up to 404 residues as the model has 404 residues like template

**Fig. 4:**
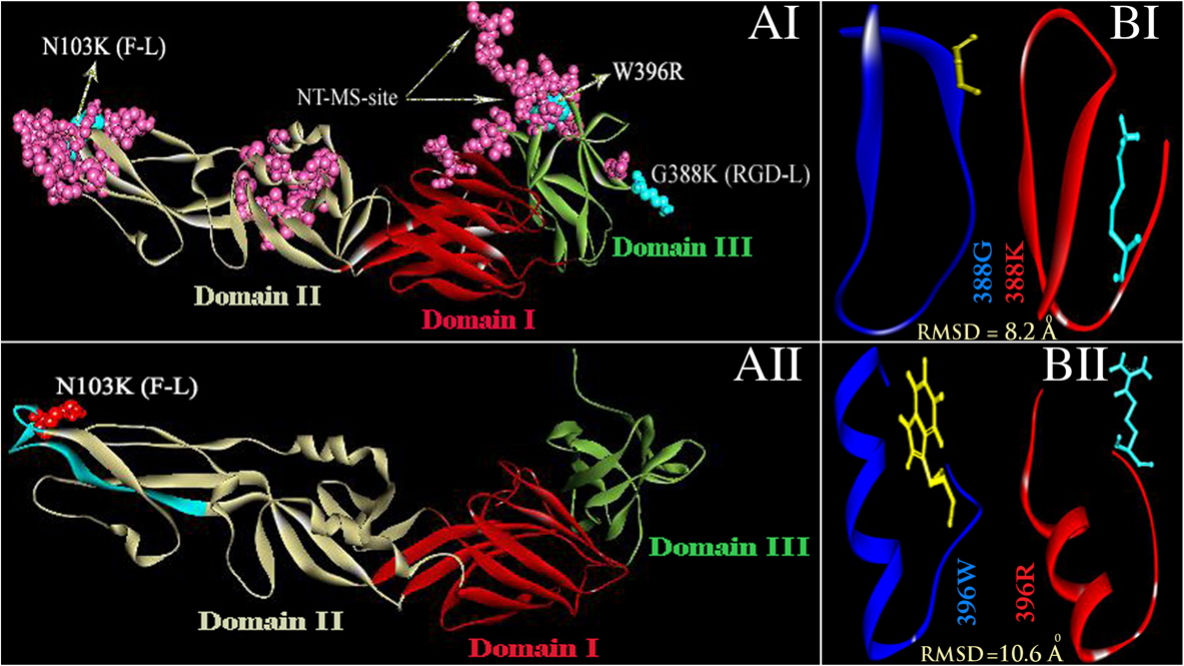
B cell specific conformational antigenic determinants (AI) and linear epitopes (AII). (AI, AII) In each case domains of E protein are presented by different colors (i.e. domain I *red*, domain II *silver* and domain III *green*). (B) Representative Epitope pairs (BI for **G388K** and BII for **W396R**) for comparison of main chain topology of wild-type (*blue*) and mutant-type (*red*). The wild-type (*yellow*) and mutant (*cyan*) type residues are shown in stick formats. Each of this peptide structure was generated using PEP-FOLD [43] followed by normalization and fixation of charge and potential. RMSD of mutant structure was computed in reference to its wild-type. F-L: Fusion loop; RGD-L: RGD loop

Linear epitopes are predicted from sequence of E protein [42]. Each epitope segment possesses specific folds or topology in three dimensional structure of the protein. We have localized all these linear epitopes in the structure of which the epitope GWG**K**GCG**L**FGKGSIDTCAKF is presented in Fig. 4 AII. It is seen that the epitope covering almost entire region of the fusion loop (residue 98–110) which is important for viral infectivity [52]. Further, it is exposed to the surface of the protein. *Ab initio* structural analyses of these epitopes are performed using PEP-FOLD method [43]. The model energy as obtained from this analysis is presented in Table 3. Negative value indicates stabilized structure (Table 3, column 4). Typical structures of selective fatal epitopes are also shown in the Fig. 4B I and II.

Although fatal mutations are seen to be populated in linear B-cell epitopes, their occurrence in conformational B-cell epitopes would be more relevant as sequentially distant residues form a three dimensional shape for binding a receptor [65]. We presented conformational epitope in Fig. 4 AI. It is seen that residues forming conformational epitopes are localized at three distinct region of E protein: two in domain II and one in domain III. The fatal mutations are seen to fall in these regions with very high specificity (≥80%). Interestingly three of four fatal mutations (**N103K, G388K** and **W396R**) are populated both in linear (Table 3) and conformational epitopes (Fig. 4 AI). Further, in the later case, mutant residue has been a part of one contiguous stretch instead of distantly and singly spaced residues coming in close proximity. Do these fatal mutations in peptide-epitopes affect overall topology? To check this we compared 3D structures of wild-type and mutant-type peptide-epitopes (Fig. 4 BI and II). It is seen that the later undergoes large conformational change relative to the earlier as revealed by their RMSD values (Fig. 4 BI and II). RMSD of 388K.pdb (MU) =8.2 Å w.r.t. 388G.pdb (WT) and RMSD of 396R.pdb (MU) =10.6 Å w.r.t. 396W.pdb (WT).

### T-cell epitope prediction

In adaptive immune response, T-cells mediated proliferation, secretion of cytokines (that stimulate antibody production by B-cells) and apoptosis are resulted when a ternary complex of (MHC-I/II)—(peptides)—(TCR-CD8^+^Tc/CD4^+^Th) is formed. T-cell-mediated immunity is essential for controlling infection of a variety of mammalian cells by flaviviruses. During viral infection, the expression of both MHC-I and MHC-II molecules increases and the functional CD8^+^T cells provide protection by direct viral clearance whereas CD4^+^T cells provide protection by eliciting protective antibody responses as well as by generating both B cell and CD8+ T cell memory responses. Flavivirus E protein is the major target of immune responses and has been shown to use both for MHC-I/CD8^+^ T cell as well as MHC-II/CD4^+^ T cell specific epitope prediction [62, 66–69]. In this study, we use IEDB Analysis Resource v2.14 [46] for prediction of T-cell epitopes.

Three score criteria were followed for screening of these peptide epitopes: a] MHC-I binding score, b] proteosomal cleavage score and c] antigenicity score. MHC-I presents endogenous peptide epitopes to the CD8^+^ cytotoxic T cell receptor (TCR). Table 4 presents predicted MHC-I (also known as HLA in human) endogenous peptides of E protein. HLA-restriction of each peptide was achieved by setting a lower cutoff for binding score (≤20) and hence better complex formation of peptide with respective allele of HLA is expected. Highly screened peptide epitopes are seen to harbor JEV genotype-I specific lethal substitutions (Table 2) which are pointed out by bold fonts with underline. The epitope YIVVGR**K**DK is most antigenic and harboring the **G388K** lethal substitution (Table 4). The alleles, with which it associates are HLA-A*03:01, HLA-A*11:01 and HLA-A*68:01 which are known to populate among Asian population [70]. Similar details are presented for other epitopes.

**Table 4:** Prediction of MHC class I specific peptide epitopes

Start	Peptide	MHC-I binding score^a^	Proteosomal Cleavage score^b^	Allele (Human Leukocyte Antigen [HLA] specific)	Antigenic Score^c^
59	Y**Y**YHASVTD	1.3–17.0	0.82	HLA-A*24:02,HLA-A*23:01,HLA-A*29:02,HLA-B*18:01,HLA-B*39:01, HLA-B*48:01,HLA-B*51:01,HLA-C*01:02,HLA-C*03:03,HLA-C*06:02, HLA-C*07:01,HLA-C*07:02,HLA-C*07:04,HLA-C*12:02,HLA-C*14:02, HLA-C*16:01,HLA-G*01:01,HLA-G*01:02,HLA-G*01:03,HLA-G*01:04, HLA-G*01:06	0.9572
95	GFTDRGWG**K**	4.4–15.5	0.85	HLA-A*03:01,HLA-A*11:01,HLA-A*29:02,HLA-A*30:01,HLA-A*33:03, HLA-A*31:01,HLA-A*68:01,HLA-A*74:01,HLA-C*04:01	1.4597
382	YIVVGR**K**DK	6.4–6.6	0.92	HLA-A*03:01; HLA-A*11:01;HLA-A*68:01	2.5185
394	HH**R**HKAGST	8.9–13.7	0.70	HLA-B*07:02, HLA-B*14:02	0.5088

^a^Percentile score vary in allele specific manner. The lower the score better is the binding specificity [46]. The range of scores was obtained by setting cut off. ^b^Proteosomal cleavage score is the average of two kinds of scores: constitutively expressed type and INF-γ induced type. ^c^Antigenic score was computed using Doytchinova and Flower's (2007) method [47]

Three score criteria were followed to screen the epitopes: MHC class I binding score, proteosomal cleavage score and antigenicity score. Association of HLA alleles for each epitope was determined by a cut off for percentile rank value ≤ 20.0. Epitopes were further checked for their efficacy in interactions at the peptide binding groove (see below)

JEV E protein was also used for prediction of MHC class II specific epitopes using the above server. MHC-II presents exogenous peptide epitopes to the CD4+ T-helper cell (Th) receptor (TCR). As MHC-II presents exogenous epitope peptides, cleavage score is not available in such prediction (Table 5). Like MHC-I, GI specific lethal substitutions are also shown in the selected peptide epitopes. Allelic association against each epitope is achieved by setting lower cutoff for MHC II binding. Lower binding score is the indicator of better complex formation between peptide epitope and MHC molecule. Highest antigenicity score and very low MHC- II binding score is observed for the epitope GFTDRGWG**K**GCGLFG which associates mostly DRB1 class of alleles of type 3, 8 and 11.

**Table 5:** MHC class II specific top scored antigenic peptides along with associated alleles

Start	Peptide	MHC-II binding score^a^	Allele (Human Leukocyte Antigen [HLA] specific)	Antigenic Score^b^
55	EVRSY**Y**YHASVTDIS	0.25–1.76	DRB1*03:05,DRB1*04:01,DRB1*04:08,DRB1*04:09,DRB1*04:07,DRB1*04:21, DRB1*04:17,DRB1*04:26,DRB1*04:62,DRB1*04:69,DRB1*04:74,DRB1*04:82, DRB1*07:03,DRB1*07:01,DRB1*08:04,DRB1*09:07,DRB1*09:04,DRB1*09:01, DRB1*09:09,DRB1*11:02,DRB1*11:21,DRB1*13:22,DRB1*15:06,DRB1*15:02	0.9127
95	GFTDRGWG**K**GCGLFG	5.37–17.95	DRB1*01:02,DRB1*03:05,DRB1*07:03,DRB1*08:01,DRB1*11:01,DRB1*11:28, DRB1*13:05,DRB1*13:21,DRB1*15:02,DRB1*15:06,DRB5*01:05	0.9232
380	DSYIVVGR**K**DKQINH	0.1–1.97	DRB1*03:06,DRB1*03:05,DRB1*03:01,DRB1*03:08,DRB1*03:07,DRB1*08:01, DRB1*08:04,DRB1*08:06,DRB1*11:01,DRB1*11:07,DRB1*13:07,DRB1*13:04, DRB5*01:01,DRB5*01:05	1.5861
383	IVVGR**K**DKQINHH**R**H	0.4–4.04	DRB1*03:06,DRB1*03:05,DRB1*03:01,DRB1*03:08,DRB1*03:07,DRB1*04:02, DRB1*08:01,DRB1*08:04,DRB1*08:06,DRB1*08:13,DRB1*11:02,DRB1*11:07, DRB1*11:21,DRB1*13:01,DRB1*13:04,DRB1*13:27,DRB1*13:22	1.1724

^a^Percentile score vary in allele specific manner. The less the score better is the binding. The range of scores was obtained by setting cut off. ^b^Antigenic score was computed using Doytchinova and Flower's (2007) method [47]

Epitopes were further checked for their efficacy in interactions at the peptide binding groove (see below)

Prediction of T-cell epitopes are performed using sequence of E protein in IEDB server [46]. How these epitopes are structurally compatible in interaction with peptide binding groove (PBG) of their corresponding MHC molecules? To check this we evaluated energetics of docked-complex of epitope. We further checked positional Shannon conservation [17, 18] and average side-chain accessibility [44] of these peptide epitopes. Table 6 presents average conservation (Table 6: column 5), accessibility (column 6), epitope conformational stability (column 7) and binding affinity for the PBG of MHCs (column 8 through 11). When positional Shannon entropy (with BLOCK length ≥70) ≤0.50, those positions are taken as conserve. In our case, observed variability for epitopes are seen to be far less and even zero (column 5). All epitopes are seen to be solvent exposed as their average accessibility is seen to be >20 (column 6). Peptides structural parameter i.e. conformational stability is seen to be negative (column 7). However, greater stability for MHC - II specific peptides seems to be related with sizes of peptide epitopes. Docking based energetics analyses are presented in column 8 through 11 (Table 6) are for overall stability (GE), Van der Waals (VDW), atomic contact energy (ACE) and hydrogen bonding (HB) interactions in Kcal Mol^−1^ respectively. All these energy of interactions are highly negative and hence stabilizing.

**Table 6:** Energetics of docking complex formed between epitopes and PBG of MHCs along with their conservation, accessibility and conformational stability values

IEDB Server Analyses				Shannon Variability	NACCESS Analysis	Peptide Analysis	Docking Analyses of MHCs-Peptides (Energy in Kcal Mol^−1^)			
	SL. NO.	Amino acid Position	Peptide	Average Shannon Entropy	Average side-chain Accessibility	CGE	GE	VDW^a^	ACE	HB
MHC-I	1	59	Y**Y**YHASVTD	0.02	23.5	−4.70	−42.89	−16.11	−5.89	−4.74
	2	95	GFTDRGWG**K**	0.03	50.4	−6.61	−28.87	−21.69	−4.29	−2.42
	3	382	YIVVGR**K**DK	0.01	32.0	−5.52	−29.36	−11.13	−4.13	−1.95
	4	394	HH**R**HKAGST	0.03	64.3	−5.84	−39.03	−14.41	−2.02	−7.97
MHC-II	1	55	EVRSY**Y**YHASVTDIS	0.04	24.7	−16.96	−48.65	−15.18	−9.98	−2.92
	2	95	GFTDRGWG**K**GCGLFG	0.02	57.9	−17.23	−46.57	−14.25	−5.75	−4.75
	3	380	DSYIVVGR**K**DKQINH	0.04	34.9	−16.07	−53.97	−22.96	−1.11	−5.02
	4	383	IVVGR**K**DKQINHH**R**H	0.00	36.8	−15.34	−52.90	−13.06	−3.63	−2.61

^a^VDW is the net stabilization due to Van der Waals interactions i.e. (attractive VDW – repulsive VDW); *GE* global stabilization energy, *ACE* atomic contact energy, *HB* hydrogen bond, *CGE* coarse-grained free-energy of peptide-structure used for docking [49]

**Fig. 5:**
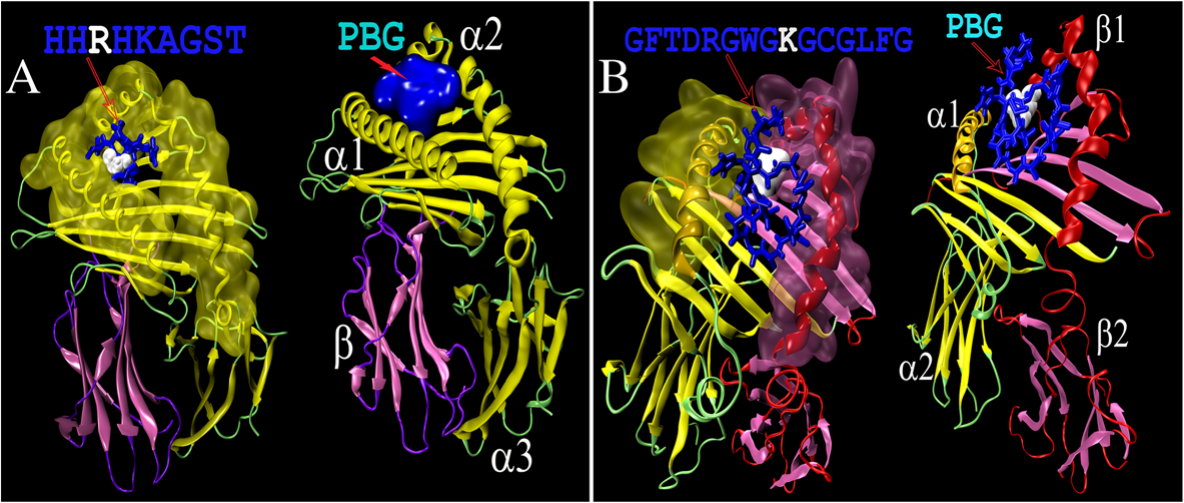
Docking and structure based short listing of antigenic epitopes using human class I MHC (HLA-A i.e. 2X4O.pdb in **a**) and class II MHC (HLA-DRB1 i.e. 1DLH.pdb in **b**) crystal structures. Typical sequence and structure of docked peptide (*blue* color A & B) with white one as mutated residue. PBG indicates peptide binding groove which was mapped using NACCESS procedure [44] (see Materials and Methods for details). Two views per MHC class are shown for better visualization of binding pocket and binding groove. Chains of both classes of MHCs are shown by conventional notations

To check the interaction of epitopes with the peptide-binding-groove (PBG) of MHC class I and MHC class II representatives, typical complexes for each class is presented in the Fig. 5. Figure shows two views from each class specific interaction along with mutated residue (white), peptide binding site (PBG). PBG is formed by two domains (α1 and α2) of α-chain (homo dimer) in MHC - I, in MHC-II it is hetero dimer and formed by α1and β1 domains of α and β chains respectively.

### Diversity in T-cell epitopes

All T-cell epitopes (Tables 4 and 5) are conserved in nature as judged by their positional Shannon Entropy values. These selected epitopes of GI strain of JEV from WB (Midnapur) and Japan harbor fatal substitutions namely: **C060Y, N103K, G388K** and **W396R**. How these epitopes are evolved in other geographical locations? To check the diversity in these epitopes a total of 613 E protein sequences were grouped into 8 geographical regions (China-228, Japan-113, Taiwan-79, India-66, Korea-35, Thailand-33, Indonesia-27 and Vietnam-32) and subjected for IEDB-Conservancy analysis. The result is presented in (Additional file 1) for MHC - I and (Additional file 2) for MHC - II epitopes. Following points are noteworthy from the observation: i] for all geographical regions and for all epitopes, large proportion of these peptide epitopes (Tables 4 and 5) remain similar as GIII type (SA14-14-2), ii] observed SNPs in these epitopes (namely: **C060Y, N103K, G388K** and **W396R**) in studied GI-strains (JEV28, JEV21 and JEV45 in WB and in Ishikawa in JAPAN) are not unique but other types of SNPs are also seen. The GI strains of Midnapur/WB/India are only examples (of all 65 Indian strains) that contain similar fatal SNPs as earlier Japan GI strain (Ishikawa), iii] these SNP harboring epitopes are mostly related with the genotypic transition from III to I and V/II. For example in China the epitope YCYHASVTD constitutes 221 WT (i.e. GIII) and 7 mutant types. Again, 6 of these mutant types are from GI and 1 is from GV, iv] frequency and position of SNP in these epitopes vary for geographical regions and v] finally GI isolates of Midnapur are seen to be similar to earlier Ishikawa/Japan type but vary greatly from other Indian and non-Indian types.

## Discussion

In spite of the availability of vaccine, JE/AES cases and deaths are frequent in different districts of West Bengal state, India. Emergence of GI and its co-circulation with GIII was reported for the first time in the district of Midnapur, WB even when patients were preimmunized with SA14-14-2 [3, 4]. Clinical symptoms and severity for GI mediated infection vary greatly from that of GIII which might involve viral and host factors. E protein plays crucial role in viral infectivity, virulence, host tropism and antigenicity [12]. In this context the reason of this genotypic transition and the study in relation to the development of GI specific vaccine are to be worked out. We apply in silico procedure using E protein sequence and its 3-dimentional models i] to identify critical among all acquired substitutions, ii] to workout epitopes harboring these critical substitutions and iii] to gain insight into the development of vaccine with potential in controlling GI cases.

### Structural properties of models

The E glycoprotein of JEV is 500 residues long of which 452 residues form extracellular domains and rest 48 residues form intra-membrane region (in part of 21, 6 and 21 residues). The protein has pI in the neutral range with net charge negative. It is predominantly β-sheeted. To gain insight into structure and mutations, we developed high precision models of GI isolates. Initial models are energy minimized to remove unwanted steric clash and to obtain global minimal structure. Multi procedure validations are done to assess quality. Low average RMSD (1.12–1.44 Å) of models indicates almost identical main chain topology. Absolute residue-surface-area and frequency of core-residues of models and template were almost identical. These assessments along with arrangement of secondary structures, specificity in di-sulfide bonds of models are indicative of well developed functional structure of the proteins. Although similar, there exists fine structural difference between models and the template. In template (from GIII) there are 33 strands of 209 residues (51%) and that for JEV28 (GI), for example, is 33 strands of 225 residues (55%). Similar difference also exists in other models which might have arisen due to genotypic variations due to substitutions (Table 1). Salt bridges are formed when side chains of acidic and basic residues are within ≤4 Å distance [71]. Similar specificity (≥75%) in these interactions in models and in template further indicates that the geometry and conformation of side chains are also well formed.

### Acquired GI specific substitutions and inducer of GIII to GI transition

GI isolates JEV28 (also JEV21) and JEV45 was discovered from the Midnapur district of WB in 2010 and 2011 respectively. These strains were isolated as co-circulation cases in patient and were seen to be capable in escaping the effect of vaccination with SA14-14-2. These patients were thus developed JE clinical symptoms [3, 4, 10, 11]. These isolates show similar locus specific mutations as Japan outbreak-GI-isolate (Ishikawa) in 1994. Our focus is to identify substitutions that may have relation with GIII to GI transition.

Mutations pertaining to neurovirulence and neuroinvasiveness seem to be genotype independent properties. These phenotypic properties depend on substitutions in E, other structural and non-structural proteins of JEV [72]. Cao QS, et al. (2011) [12] showed that the sequence of E protein of their GI isolate is identical as virulent GIII strain (SA14) but differ from SA14-14-2 at 8 different positions (**F107L, K138E, V176I, A177T, H264Q**, **M279K, V315A** and **R439K**) which they logically considered to be responsible for virulence phenotype of their isolate. Exactly similar results were also obtained both for GIII (JEV46 and JEV47) and GI (JEV28 and JEV45) isolates [4] indicating the observation is genotype independent. It is noteworthy that E protein of virulent (SA14) and non-virulent (SA14-14-2) strains differ by first 6 mutations (Table 1, marked with ^a^ sign) [73]. Neuroinvasiveness could largely be related with the substitutions in other proteins but not in E protein. Nerome R, et al. (2007) and Cao QS, et al. (2011) [12, 13] reached to the same conclusion as far as neuroinvasiveness is concerned. Moreover, in reference to GP78/India [1] it was concluded that SA14-14-2 needs **E244G, A315V** and **S366A** for complete neurovirulence [72]. Nevertheless crucial substitutions that cause the transition of existing GIII to newly emerged GI (JEV28 and JEV45) in Midnapur district and allow escaping the effect of vaccination remained unanswered.

Table 1 compares substitutions in JEV28 and JEV45 (also JEV21; Ishikawa/Japan/1994) with non-virulent (SA14-14-2) vaccine strain (also virulent SA14; PDB ID 3P54). As mentioned above, mutations at positions: **138, 176** and **177** (in DI) and **107, 279** and **264** (in DII) emerge due to attenuation of virulent phenotype. SA14 and SA14-14-2 also differ at these positions (Table 1, mutations marked with^a^ sign). Additionally the ecto domain needs **G244E, V315A** and **A366S** substitutions for virulent phenotype (see above). It therefore indicates that these mutations are neither related to the transition of GIII to GI nor with the escape of vaccine effects. Thus to gain insight into the effect of homologous substitutions that may induce emergence of GI, we attempted rigorous sequence [37–40] and structure [41] based approaches using sequence and structure as input respectively. Out of all substitutions, only **C60Y, N103K, G388K** and **W396R** are GI specific and disease associated. As **N103K** is the only one which is common in all our GI isolates and showing disease association, it might be crucial for GIII to GI transition and escaping of GIII vaccine effect. It is present in the fusion loop region (98–110) which is crucial for viral infectivity [52, 59]. JEV45 (also JEV21) contains two additional GI specific fatal substitutions (**G388K** and **W396R)** that are absent in JEV28. **C60Y** is only present in Ishikawa strain. In silico site directed free energy evaluation [58] shows that **C60Y** and **W396R** are destabilizing whereas **N103K** and **G388K** are stabilizing. **C60Y** destroys one conserved disulfide bond (between ***C***_***60***_ ***– C***_***116***_) and **W396R** causes removal of conserved Tryptophan residue. The stabilizing effect of **N103K** and **G388K** might be due to the side chain of Lysine that possesses a long flexible hydrocarbon tail with positive charge at its end which might facilitates stabilizing electrostatic interactions. Further G at **388** of DIII/E protein is present in the **RGD** motif [6, 60] important for cell-cell interaction. Substitution of such conserved glycine that imparts flexibility is known to have worst structural effect [74]. Overall, these 4 SNPs that are acquired in the course of viral evolution seems to be crucial for GI specific characteristics of E protein of which **N103K** acts as initiator for transition.

Other substitutions such as **N2H, N2T, T129M, A177T, Y183F, R193K, A222S, G244E, G261S, H264Q, V315A, S327T, A366S, V372L** and **M374I** are non-disease associated. They could be divided into two categories: neutral and non-neutral-non-disease associated. While the former do not affect conformational properties of mutant protein the later does to some extent.

### B-cell epitopes harboring fatal substitutions and are crucial for neutralizing antibody

Major challenge remains to understand the escape of neutralization of GI specific infection upon vaccination [3, 4]. The purpose of vaccination is to induce proliferation of neutralizing antibody that in turn is expected to neutralize viral infection. The reports of co-circulation cases (GIII with GI in patients) in the district of Midnapur/WB have been a serious concern. In this end the substitutions **F107L, K138E, V176I, A177T, H264Q, M279K, G244E, V315A** and **A366S** are unlikely to be responsible as i] these are related with virulent phenotype and ii] are observed both with GI and GIII isolates [4]. Prediction of neutralizing epitopes harboring fatal SNPs would not only help to understand escape mechanism but also be useful in designing GI specific vaccine that might take care possible GI-mediated outbreak in the region in near future. Our studies shows that of all SNPs only 4 such as **C60Y, N103K, G388K** and **W396R** are disease-associated of which former two are in domain-II and the later two are in domain-III of E protein which are the source of major antigenic determinants [53]. The question that are these SNPs present in our predicted population of B-cell epitopes. B-cell epitopes, both configurational (linear) and conformational (spatial), are thus predicted using authentic web servers. Initial population of both these epitopes is screened based on their scores. Linear epitopes were further screened for: a] accessibility, b] epitopes model energy and c] average conservation properties. It is interesting to note that these highly screened B-cell epitopes (Table 3) harbor disease associated SNPs namely **C60Y, N103K, G388K** and **W396R**. Of these four, **N103K** in fusion loop, **G388K** in **RGD** loop and **W396R** is in the N-terminal membrane attachment site (NT-MS site) (Fig. 4 AI). These regions are known to play important role for antigenicity and infectivity [75]. Their presence in both linear and conformational epitopes (in contiguous stretch of residues) indicates their importance in the development of vaccine. Large variations of RMSD of GI specific lethal mutation harboring peptide epitopes, point to the shift of wild-type (GIII specific) specificity in the neutralization reaction.

The only lethal substitution **N103K** is present in Midnapur/WB/JEV28 whereas Midnapur/WB/JEV21 and Midnapur/WB/JEV45 contain **N103K, G388K** and **W396R**. All these three isolates of Midnapur are newly emerged as GI [3]. Sarkar et al. (2013) interpreted the mutation **G388K** to be responsible “to escape from antibody neutralization or neutralizing epitope” based on physiochemical property and IC_50_ score for MHC-I specific T-cell epitopes without confirming the presence of the residue in B-cell epitopes. In fact B-cell epitopes were not determined in the work. The authors have applied similar approach for the substitution **N103K**. The fact that substitutions at homologous positions could be acquired either by natural selection that affects protein function [76] or by genetic drift that imparts neutral effect [77] understanding the effects of substitutions on protein function need careful criteria-based evaluation. It may further be essential to confirm the presence of these substitutions on epitope for understanding escape mechanism. However, apart from the above linear epitopes, conformational B-cell epitopes (Fig. 4 AI) which are seen to be localized in most antigenic domains (DII and DIII) of the protein are seen to contain these above mentioned fatal SNPs with very high scores. The fact that each of these SNPs is seen to affect overall conformational stability of mutated E protein (Table 2) the possibility of altered specificity in neutralization reaction could not be ruled out. Taken together, it could be said that observation of repeated co-infection cases [3, 4] might be due to altered specificity of antigenic determinants harboring these fatal SNPs. These epitopes would also be useful for designing GI specific vaccine.

### Fatal substitutions in T-cell epitopes

T-cell mediated immune response plays crucial role in proliferation of both cytotoxic T-lymphocytes and cytokines induced B-cell antibodies [78]. Length of MHC class I specific epitopes are 8 to 11 amino acids long and that for MHC class II is 13 to 17 amino acids long [78]. Unlike B-cell epitopes, selection of T-cell ones are MHC classes and alleles specific. We selected only four epitopes from each class following strict cutoff for MHC binding, proteosomal cleavage (for MHC class I only) and antigenicity score. Of all MHC class I epitopes, **YIVVGR** **K** **DK** possesses highest antigenicity and proteosomal cleavage score. It contains the lethal substitution **G388K** in its **RGD** motif which is known for host cell interaction [6, 60]. The substitution, present in Midnapur (JEV45 and JEV21) and Japan isolates (Ishikawa), is shown to affect overall conformational stability of E-protein. Thus it may affect T-cell proliferation and cytokine mediated B-cell antibodies production. Interestingly the same substitution **G388K** is also present in MHC class II specific epitope **DSYIVVGR** **K** **DKQINH** with very high binding and antigenicity score. The first GI isolate (JEV28) from Midnapur/WB contains only one lethal substitution i.e. **N103K**. It is present in the fusion loop of the E protein which is important for viral infectivity. Our predicted MHC class (I and II) specific epitopes has been **GFTDRGWG** **K** and **GFTDRGWG** **K** **GCGLFG** respectively that contains the said mutation. The former has higher specificity (lower binding score and higher antigenicity score). The fact that the patients, from which these strains were isolated, were reported to escape the neutralizing effect of GIII specific vaccine [4], these set of B-cell (see above) and T-cell epitopes can be used as reference for development of GI specific vaccine. Moreover other predicted epitopes that harbor lethal substitutions (such as **C60Y** and **W396R**) are also potential to alleviate GI specific danger [3, 4] from the district of Midnapur (see below).

Major (A, B, C) and minor (G) class I type HLA alleles that are associated with our selected epitopes are major alleles for zoonotic viral infection [79]. Similarly, haplotypes DRB1*04, DRB1*03, DRB1*08, DRB1*09 and DRB1*11 are abundant in our class II specific epitopes. These haplotypes were seen to be associated with zoonotic viral infection [80, 81].

The selected B-cell and T-cell epitopes of GI isolates of Midnapur/WB (JEV28, JEV21 and JEV45) are compared with isolates from other geographical regions such as China, Japan, Taiwan, Korea, Thailand, Vietnam and Indonesia (see Additional files 1 and 2). The mutation patterns of these epitopes are seen to be similar as Ishikawa/Japan but differ for isolates from other geographical regions. The mutated populations of these epitope-segments are very low (≤2%) and are mostly related with genotypic transition from GIII to GI/GV/GII. For example of 228 isolates from China for the epitope **YCYHASVTD**, 220 are of GIII type, 7 are GI and 1 is GV type (see Additional file 1). Epitope-segments occupy different parts of E protein sequence that may have differential selection pressure from different geographical regions. For example the epitope **YIVVGRGDK** (382–390) shows more variants in Vietnam (7 of 32) than China (4 of 228) isolates and no variations are seen in Thailand isolates (0 in 33).

## Conclusions

Our studies employ wide range of sequence and structure based in silico procedures on E protein of GI isolates from Midnapur/WB to understand GI specific lethal substitutions, its transition from the pool of GIII strain of JEV and prediction of B-cell and T-cell epitopes along with their diversity in Asian continent. Nine substitutions are commonly seen in the ecto domain of E protein of GI and GIII isolates are largely act as determinant of virulent phenotype of these GI isolates. 4 substitutions namely **C60Y, N103K, G388K** and **W396R** are lethal and GI specific of which former two are in domain II and the later two are in domain III. The lethality largely related to the overall destabilization of the protein, its malfunctioning and disease association. These four substitutions affect most conserved region of the E protein. While in domain II, **C60Y** destroys a conserved di-sulfide bond, **N103K** occurs in the highly conserved fusion loop. **G388K** disrupts conserved **RGD** motif and **W396R** destroys most conserved tryptophan residue in domain III. **N103K**, the only lethal mutation which is common among all three GI/WB isolates (JEV28, JEV21 and JEV45) and earlier Ishikawa/JAPAN isolate, could be the reason for genotypic transition from existing GIII to newly emerge GI isolate in the region. Highly antigenic, top scored B-cell (both linear and conformational) and T-cell (both HLA class I and II) epitopes are seen to harbor these critical substitutions and thus these subset of epitopes may act as reference for design of GI specific vaccine. Although mutation pattern vary, these subset of epitopes act as target site for acquisition of genotypic transition related substitutions for other isolates of Asian continent. Overall our approaches highlight detailed aspects of immunoinformatics and find applications in the context of other infectious disease causing systems.

## Additional files

Additional file 1: Diversity in MHC class I specific epitopes in isolates from 8 different Asian countries. Analysis was performed using IEDB resources [46] against the ecto domain of E protein that were procured from GenBank databases. Partial or fragmented sequences were excluded. (DOCX 14 kb)
Additional file 2: Diversity of MHC class II specific epitopes in isolates discovered from 8 different Asian countries. Analysis was performed using IEDB resources [46] against the ecto domain of E protein that were procured from GenBank databases. Partial or fragmented sequences were excluded. (DOCX 14 kb)

## Abbreviations

ACE: Atomic contact energy
AES: Acute encephalitis syndrome
ASA: Accessible surface area
C: Capsid C
CGE: Coarse-grained free-energy
CHARMM: Chemistry at HARvard macromolecular mechanics
DOPE: Discrete optimized protein energy
E protein: Envelope protein
EMBOSS: European molecular biology open software suite
GE: Global stabilization energy
GI: Genotype I
GIII: Genotype III
GRAVY: Grand average hydropathy
HB: Hydrogen bonding
HLA: Human leukocyte antigen
HMM-SA: Hidden Markov Model-derived Structural Alphabet
JE: Japanese encephalitis
JEV: Japanese encephalitis virus
MHC: Major histocompatibility complex
MU: Mutant
NAMD: Nanoscale molecular dynamics
NT-MS site: N-terminal membrane attachment site
OPEP: Optimized potential for efficient protein structure prediction
PBG: Peptide-binding-groove
PDB: Protein data bank
PMDB: Protein model data base
prM: Precursor membrane
RMSD: Root mean square deviation
SDM: Site directed mutator
SNPs: Single nucleotide polymorphisms
TCR: T-cell receptor
Th: T-helper cell
UP: Uttar Pradesh
VDW: Van der waals
VMD: Visual molecular dynamics
WB: West Bengal
WT: Wild-type
