# Supplementary material for: Insight into SNPs and epitopes of E protein of newly emerged genotype-I isolates of JEV from Midnapur, West Bengal, India
Source: BMC Immunol. 2017 Mar 6;18:13. doi: 10.1186/s12865-017-0197-9 (PMC5339996; doi:10.1186/s12865-017-0197-9)
Supplement: Additional file 1: — Diversity in MHC class I specific epitopes in isolates from 8 different Asian countries. Analysis was performed using IEDB resources [46] against the ecto domain of E protein that were procured from GenBank databases. Partial or fragmented sequences were excluded. (DOCX 14 kb) [file 12865_2017_197_MOESM1_ESM.docx]

**Additional File 1: Diversity in MHC class I specific epitopes in isolates from 8 different Asian continents. Analysis was performed using IEDB resources [46] against the ecto domain of E protein that were procured from GenBank databases. Partial or fragmented sequences were excluded.**

| **Peptide** | **China**  **Isolates=228** | **Japan**  **Isolates=113** | **Taiwan**  **Isolates=79** | **India**  **Isolates=66** | **Korea**  **Isolates=35** | **Thailand**  **Isolates=33** | **Vietnam**  **Isolates=32** | **Indonesia**  **Isolates=27** |
| --- | --- | --- | --- | --- | --- | --- | --- | --- |
| **YYYHASVTD**  **59** | **YCYHASVTD220**  **-Y-------1**  **---R-----2**  **------A--1**  **--------H1**  **-------P-1**  **H--------1**  **-----T-A-1** | **YCYHASVTD109**  **-Y-------1**  **---R-----1**  **-------I-1**  **-------N-1** | **YCYHASVTD77**  **---R-----1**  **------I--1** | **YCYHASVTD65**  **-----T---1** | **YCYHASVTD29**  **-------S-1**  **-----L---1**  **-----T-A-4** | **YCYHASVTD32**  **---Y-----1** | **YCYHASVTD31**  **------I--1** | **YCYHASVTD25**  **--H------1**  **------I--1** |
| **GFTDRGWGK**  **95** | **GFTDRGWGN225**  **--------K2**  **-Y-------1** | **GFTDRGWGN111**  **--------K1**  **--A------1** | **GFTDRGWGN78**  **-------R-1** | **GFTDRGWGN59**  **--------K7** | **GFTDRGWGN31**  **-Y-------4** | **GFTDRGWGN33** | **GFTDRGWGN29**  **----P----1**  **-V-------2** | **GFTDRGWGN27** |
| **YIVVGRKDK**  **382** | **YIVVGRGDK224**  **---------**  **-----TR--1**  **---I-----1**  **-----M---1**  **F--------1** | **YIVVGRGDK112**  **------K--1** | **YIVVGRGDK74**  **-----M---1**  **-------H-1**  **-------G-2**  **-------N-1** | **YIVVGRGDK59**  **------K--2**  **-------E-3**  **------E--1**  **--E------1** | **YIVVGRGDK30**  **F--------4**  **--E------1** | **YIVVGRGDK33** | **YIVVGRGDK25**  **--------F1**  **--------E1**  **--E------1**  **-----I---1**  **------V--1**  **-V-------1**  **---G-M---1** | **YIVVGRGDK23**  **-------E-2**  **-----E---1**  **-X-------1** |
| **HHRHKAGST**  **394** | **HHWHKAGST220**  **---Y-----6**  **--------M1**  **--------S1** | **HHWHKAGST111**  **--R------1**  **Q--------1** | **HHWHKAGST75**  **---Y-----2**  **--------K2** | **HHWHKAGST55**  **--R------2**  **----R----1**  **---Y-----3** | **HHWHKAGST29**  **--------S4**  **---Y-----1**  **-------K-1** | **HHWHKAGST28**  **---Y-----4**  **-----T---1** | **HHWHKAGST31**  **---P---N-1** | **HHWHKAGST22**  **-----P---4**  **-----T---1** |
