# Supplementary material for: Insight into SNPs and epitopes of E protein of newly emerged genotype-I isolates of JEV from Midnapur, West Bengal, India
Source: BMC Immunol. 2017 Mar 6;18:13. doi: 10.1186/s12865-017-0197-9 (PMC5339996; doi:10.1186/s12865-017-0197-9)
Supplement: Additional file 2: — Diversity of MHC class II specific epitopes in isolates discovered from 8 different Asian countries. Analysis was performed using IEDB resources [46] against the ecto domain of E protein that were procured from GenBank databases. Partial or fragmented sequences were excluded. (DOCX 14 kb) [file 12865_2017_197_MOESM2_ESM.docx]

**Additional File 2: Diversity of MHC class II specific epitopes in isolates discovered from 8 different Asian continents. Analysis was performed using IEDB resources [46] against the ecto domain of E protein that were procured from GenBank databases. Partial or fragmented sequences were excluded.**

| **Peptide** | **China**  **Isolates=228** | **Japan**  **Isolates=113** | **Taiwan**  **Isolates=79** | **India**  **Isolates=66** | **Korea**  **Isolates=35** | **Thailand**  **Isolates=33** | **Indonesia**  **Isolates=27** |
| --- | --- | --- | --- | --- | --- | --- | --- |
| **EVRSYYYHASVTDIS**  **55** | **EVRSYCYHASVTDIS217**  **-----Y---------1**  **K--------------1**  **------------H--1**  **---R-----------1**  **-------R-------2**  **----H----------1**  **-----------P---1**  **----------A----1**  **-I-------------1**  **---T-----T-A---1** | **EVRSYCYHASVTDIS109**  **-----Y---------1**  **-------R-------1**  **-----------I---1**  **-----------N---1** | **EVRSYCYHASVTDIS77**  **----------I----1**  **-------R-------1** | **EVRSYCYHASVTDIS65**  **---------T-----1** | **EVRSYCYHASVTDIS28**  **D--------------1**  **---T-----T-A---3**  **---A-----T-A---1**  **-----------S---1**  **---------L-----1** | **EVRSYCYHASVTDIS32**  **-------Y-------1** | **EVRSYCYHASVTDIS25**  **------H--------1**  **----------I----1** |
| **GFTDRGWGKGCGLFG**  **95** | **GFTDRGWGNGCGLFG207**  **--------K------2**  **-------------Y-1**  **----------W----1**  **-----------F---14**  **-----------R---1**  **--------------R1**  **-Y-------------1** | **GFTDRGWGNGCGLFG111**  **--------K------1**  **--A------------1** | **GFTDRGWGNGCGLFG78**  **-------R-------1** | **GFTDRGWGNGCGLFG59**  **------K--------7** | **GFTDRGWGNGCGLFG28**  **-Y-------------4**  **------------F--3** | **GFTDRGWGNGCGLFG33** | **GFTDRGWGNGCGLFG26**  **-------------S-1** |
| **DSYIVVGRKDKQINH**  **380** | **DSYIVVGRGDKQINH224**  **-----I---------1**  **-------M-------1**  **-------TR------1**  **--F------------1** | **DSYIVVGRGDKQINH110**  **--------K------1**  **------------V--1**  **--------------Q1** | **DSYIVVGRGDKQINH74**  **-------M-------1**  **---------H-----1**  **---------G-----2**  **---------N-----1** | **DSYIVVGRGDKQINH58**  **--------K------2**  **---------E-----3**  **--------E------1**  **-------------S-1**  **----E--------S-1** | **DSYIVVGRGDKQINH29**  **G--------------1**  **--F------------4**  **----E----------1** | **DSYIVVGRGDKQINH33** | **DSYIVVGRGDKQINH23**  **---------E-----2**  **-------E-------1**  **---X-----------1** |
| **IVVGRKDKQINHHRH**  **383** | **IVVGRGDKQINHHWH219**  --------------Y6  **----TR---------1**  **----M----------1**  **--I------------1** | **IVVGRGDKQINHHWH110**  **-----K-------R-1**  **---------V-----1**  **-----------Q---1** | **IVVGRGDKQINHHWH72**  **--------------Y2**  **------G--------2**  **------N--------1**  **------H--------1**  **----M----------1** | **IVVGRGDKQINHHWH55**  **-------K-----R-2**  **------E--------3**  **----------S----1**  **--------------Y3**  **-E--------S----1** | **IVVGRGDKQINHHWH33**  **--------------Y1**  **-E-------------1** | **IVVGRGDKQINHHWH29**  **--------------Y4** | **IVVGRGDKQINHHWH23**  **------E--------2**  **----E----------1**  **X--------------1** |
